# Influence of Vineyard Inter-Row Management on Grapevine Leafhoppers and Their Natural Enemies

**DOI:** 10.3390/insects15050355

**Published:** 2024-05-14

**Authors:** Elena Cargnus, Seyedeh Fatemeh Kiaeian Moosavi, Davide Frizzera, Chiara Floreani, Pietro Zandigiacomo, Giovanni Bigot, Davide Mosetti, Francesco Pavan

**Affiliations:** 1 Department of Agricultural, Food, Environmental and Animal Sciences, University of Udine, Via delle Scienze 206, I-33100 Udine, Italy; seyedehfatemeh.kiaeianmoosavi@uniud.it (S.F.K.M.); davide.frizzera@uniud.it (D.F.); floreanichiara817@gmail.com (C.F.); pietro.zandigiacomo@uniud.it (P.Z.); francesco.pavan@uniud.it (F.P.); 2 Perleuve S.r.l., Via Isonzo 25/1, I-34071 Cormons, Italy; giovanni@perleuve.it; 3 Independent Researcher, Via Cividale 55, I-34072 Gradisca d’Isonzo, Italy; davide.mosetti@gmail.com

**Keywords:** Araneae, alternate mowing, *Anagrus atomus*, egg parasitoid, *Hebata vitis*, spiders, tillage, vine vigor, *Zygina rhamni*

## Abstract

**Simple Summary:**

The inter-row management strategy in the vineyard influences the diversity of the vegetation and, consequently, the presence and activity of natural enemies of grapevine pests. This study aims to evaluate whether the replacement of periodical tillage with the alternate mowing of the resident herbaceous vegetation in vineyard inter-rows reduces the density of the European leafhoppers *Hebata vitis* and *Zygina rhamni* by improving the presence and activity of their natural enemies in the context of conservation biological control. The results show that, on grapevines with ground vegetation, the number of hunting spiders increases, thus reducing the leafhopper population with their predation activity. Green covering also improves hymenopteran parasitoids characterized for their important role in the grapevine pest control. A greater vegetation diversity in the vineyard improves the landscape quality of the agroecosystem and, by supporting the activity of the natural enemies of grapevine pests, reduces the need for insecticide applications in the vineyards.

**Abstract:**

Inter-row management in vineyards can influence the abundance of grapevine pests and their natural enemies. In 2013–2015, in a vineyard in northeastern Italy, the influence of two vineyard inter-row management strategies (i.e., alternate mowing, AM, and periodical tillage, PT) on the population dynamics of grapevine leafhoppers *Hebata vitis* and *Zygina rhamni* and their natural enemies, the mymarid *Anagrus atomus* and spiders (Araneae), and other hymenopteran parasitoids, were studied with different survey approaches. The infestations of both leafhoppers were lower in AM than PT due to the reduced leafhopper oviposition and higher nymph mortality in AM. This occurred although leafhopper egg parasitization by *A. atomus* was greater in PT than AM according to a density-dependent relationship with the leafhopper egg amount. Hymenopteran parasitoids other than *A. atomus* were the most abundant in AM, probably due to the higher availability of nectar and pollen than in PM. The significantly higher population densities of hunting spiders in AM than PT can be associated with the higher predation of leafhopper nymphs. Therefore, the study demonstrated that the alternate mowing of vineyard inter-rows enhances the abundance of natural enemies, such as spiders and hymenopteran parasitoids, and can contribute to grapevine leafhopper pest control.

## 1. Introduction

*Hebata vitis* (Göthe) [1] [syn. *Empoasca vitis* (Göthe)] and *Zygina rhamni* Ferrari (Hemiptera: Cicadellidae) are the most widespread leafhoppers in European vineyards [2]. *Hebata vitis* overwinters as adults on evergreen plants and can complete one to four generations a year [3,4,5,6]. The feeding activity on leaf veins causes redness or yellowing, starting from the leaf margins, followed by drying [4,7]. The associated physiological damage [8] can lead to yield losses and a decreased sugar content in the berries [9,10,11,12]. The leafhopper *Z. rhamni* overwinters at the egg and adult stages mostly on *Rubus* spp. on which it can complete a generation in early spring before migrating towards vineyards where it has three generations per year [4,13,14]. It is a mesophyll feeder that rarely reaches harmful numbers [4,15]. In some areas of north-eastern Italy, economic damage has been recently reported for *Erasmoneura vulnerata* (Fitch) (Hemiptera: Cicadellidae), a mesophyll-feeding leafhopper introduced in the early 2000s from North America [16,17].

Previous studies showed that inter-row management based on ground cover improves the grapes’ quality and reduces bunch roots [18,19,20].

As part of sustainable solutions for the leafhopper control in vineyards, the alternative management of vineyard inter-rows could provide potential benefits in both organic vineyards where synthetic pesticides are prohibited and conventional vineyards where the restriction on the agrochemical use is increasing. Ground vegetation can provide shelters, alternative prey or hosts, and food (i.e., nectar, honeydew, and pollen) to several beneficials [21,22,23], improving the biological control of grapevine pests [24,25,26,27,28,29].

In European vineyards, the main natural enemy of *H. vitis*, *Z. rhamni*, and *E. vulnerata* is the egg parasitoid *Anagrus atomus* L. (Hymenoptera: Mymaridae) (*sensu* Triapitsyn et al. [30]) [17,31,32,33,34,35]. This parasitoid overwinters mostly in leafhopper eggs laid on *Rubus* spp. and *Rosa* spp. leaves, and the removal of these plants from the vineyard ecosystem is thought to be one of the factors that favored *H. vitis* infestations in vineyards [34,36]. However, no data are available on the effect of the alternative vineyard inter-row management on the *H. vitis* parasitization by *A. atomus*. Among predatory arthropods inhabiting vineyards, spiders (Araneae) are the most abundant (up to 98%) [37], and their occurrence in vineyards is investigated worldwide [29]. Although spiders are reported as predators of leafhoppers in European vineyards [38,39], no studies investigated the role of ground vegetation on the grapevine leafhopper’s predation by spiders. Specifically, inter-row tillage in vineyards of north-eastern Italy is usually adopted to reduce the water and nutrient competition between grapevines and herbaceous plants. However, this inter-row management is claimed to favor infestation by leafhoppers and other grapevine pests. This phenomenon, if demonstrated, could be due to the absence of suitable habitats for their natural enemies [40,41,42] or could be the consequence of effects on the vegetative status of grapevines (i.e., vigor and water stress) [43,44,45,46]. In particular, it has been shown that more vigorous grapevines are associated with greater populations of *H. vitis* [43,44], and moderate water stress, which could also be favored by the presence of herbaceous vegetation in the inter-rows, reduces the nymph population of some leafhoppers due to a lower rate of egg hatching from leaf veins with poor water availability [45,46,47]. It was demonstrated that nectar and pollen from herbaceous plants are favorable to the presence of adults of hymenopteran parasitoids [27,40]. In vineyards, these last were assessed for their potential role in the control of carpophagous and leaf-miner Lepidoptera infesting grapevines [22,48,49,50,51,52,53]. Therefore, this study was aimed at evaluating the effect of the substitution of the usual inter-row tillage with the alternative inter-row mowing, maintaining every second row with the presence of herbaceous flowering plants, on (i) the population density of grapevine leafhoppers, (ii) the activity of their natural enemies, *A. atomus* and spiders, and (iii) the abundance of other hymenopteran parasitoids. Inter-row management strategies that promote biodiversity, although encouraged by our region (Friuli Venezia Giulia, north-eastern Italy) also through public subsidies, have not always been adopted by winemakers because they fear a reduction in yield. In this context, we wanted to verify whether the alternate mowing could have a positive effect on the control of grapevine leafhoppers. An secondary aim of this study was to evaluate whether the cultivar clone can interfere with the effect of inter-row management on the abundance of grapevine leafhoppers and natural enemies.

## 2. Materials and Methods

### 2.1. Study Vineyard

**Figure 1:**
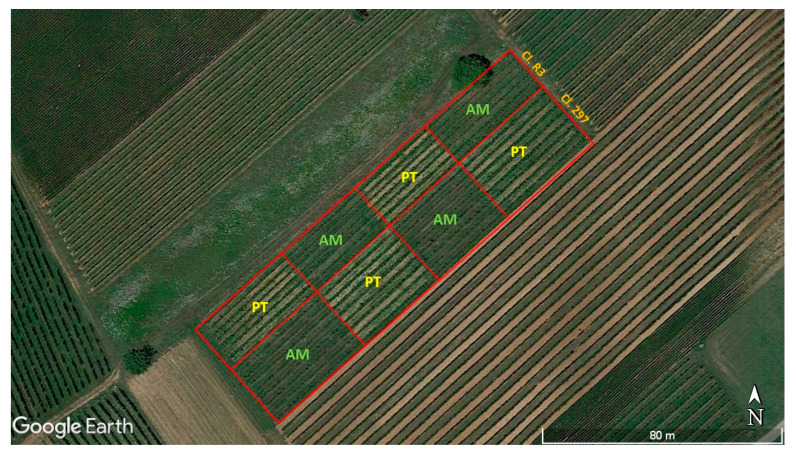
Experimental design adopted in the study vineyard, planted with two clones (Cl. 297 and Cl. R3) of Sauvignon Blanc, with alternate mowing (AM) and periodical tillage (PT) of inter-rows in comparison. The plots are delimited with the red lines (modified from Google Earth).

The study was conducted for three consecutive years (2013–2015) in an organic vineyard in northeastern Italy (Cormons, Gorizia, 45°56′ 56″ N, 13°27′11″ E, 46 m a.s.l.) (Figure 1). The vineyard, planted in 1980 with Sauvignon Blanc (*Vitis vinifera* L.), had 16 grapevine rows 140 m long and N48° E orientated. The grapevines grafted on SO4 rootstock were spaced 1.7 m within rows and 2.7 m between rows, and grown with a double-Guyot training system. The first nine rows from the south side were planted with cultivar clone 297 and the following seven with cultivar clone R3 (hereafter, “cultivar clone” is referred to as “clone”). In the years before the study, every second inter-row was periodically tilled. Other vineyards, field crops and spontaneous meadows surrounded the vineyard. No insecticides and only sulfur- and copper-based fungicides were applied to grapevines during the three-year study.

### 2.2. Inter-Row Management Strategies

From early spring 2013, two inter-row management strategies were established in the vineyard: alternate mowing and periodical tillage (Figure 1). In the alternate mowing treatment (hereafter, “AM”), every second inter-row was, in turn, mowed when flowering began in the contiguous inter-row (mostly Apiaceae, Asteraceae, and Fabaceae; plant species reported in Table S1 in the Supplementary Materials). This management technique was adopted to maintain flowering plants on at least one side of each grapevine row. Thus, nectar and pollen production as a food supply for natural enemies was achieved throughout the vegetative season. In the periodical tillage treatment (hereafter, “PT”), the soil of the inter-rows was cultivated to remove herbaceous plants. To achieve this aim twice per year (6 May and 1 July 2013; 5 May and 5 July 2014; and 8 May and 7 July 2015), inter-rows of the AM were mowed, and those of the PT were tilled, using a shredding machine and a tractor-mounted tiller, respectively.

Each inter-row treatment was replicated in two plots for each clone (Figure 1). Plot size was 550 m^2^ (around 120 vines) for clone R3 and 750 m^2^ (around 160 vines) for clone 297.

Meteorological data were obtained from a weather station (Gradisca d’Isonzo, Gorizia, ARPA-OSMER http://www.osmer.fvg.it/ (accessed on 15 February 2023)) located 6 km from the study vineyard (Table S2 in the Supplementary Materials).

### 2.3. Samplings

From 2013 to 2015, the following arthropod taxa were sampled: grapevine leafhoppers (Hemiptera: Cicadellidae), *Anagrus atomus*, Ichneumonoidea, Chalcidoidea excl. Mymaridae, and spiders (Araneae).

Nymphs of grapevine leafhoppers were sampled in the field by visual inspection of leaves every two weeks, from 19 June to 27 August 2013 (6 samplings), from 20 May to 25 August 2014 (8 samplings), and from 20 May to 26 August 2015 (8 samplings). At each sampling date, 100 mature grapevine leaves per plot (400 leaves per treatment) were observed in the central row, excluding the five plants next to the plot edge. Nymphs of *H. vitis* and *Z. rhamni* were counted and identified after Pavan et al. [54]. The possible presence of the recently introduced *E. vulnerata* was ascertained based on the description reported in Duso et al. [55]. In 2013, samplings started only in late June because, in previous sampling dates (i.e., 21 May, 4 June, and 18 June), no leafhopper nymphs were detected after observing 100 leaves per treatment.

All arthropod taxa studied were monitored by yellow sticky traps [24 × 12 × 0.2 cm, smeared with glue (Temo-O-Cid^®^, Kollant Srl, Vigonovo, Venezia, Italy)]. These traps were hung on the horizontal wires of the grapevine trellis at about 1.5 m from the ground level. After their first installation (9 May 2013, 6 May 2014, and 8 May 2015), the traps were replaced approximately every two weeks until harvest time (27 August 2013, 25 August 2014, and 11 August 2015), for a total of 9, 9, and 8 sampling periods in the three years, respectively. Two traps per plot (eight traps per treatment) were placed along the central row, respecting a distance of 15 m between the traps and 10 m from the edge of the row. In the laboratory, the captures of arthropods were counted and taxonomically sorted under a dissection microscope. Adults of grapevine leafhoppers and *A. atomus* were identified [4,30,55,56,57]. Ichneumonoidea and Chalcidoidea excl. Mymaridae were distinguished based on the morphological features of wings [58]. Spiders were counted without specifying further taxonomic level and hunting strategy.

Spider populations in the grapevine canopy were also assessed by a drop-cloth method. They were sampled on the same dates as the yellow sticky trap installation and replacement, except in one sampling in early June 2013 (i.e., 8, 9, and 8 samplings in 2013, 2014, and 2015, respectively). Drop-cloth consisted of manually shaking a grapevine canopy five times, grabbing the apical part of the trunk, and collecting fallen spiders on a pale cloth sheet (74 × 45 cm). At each sampling date, the spiders collected on 10 grapevines per plot (40 grapevines per treatment) (5 in the central row and 5 on an adjacent row) were separately preserved in vials with 70% aqueous ethanol. Spiders were identified at the family level and, when possible, at genus or species levels [59]. Based on family, spiders were grouped as web-builders and hunters.

To count the leafhopper eggs, just before the harvest (4 September 2013, 9 September 2014, and 20 August 2015), 20 mature grapevine leaves per plot (80 leaves per treatment) were collected in the central row, excluding the five plants next to the plot edge, and enclosed in a plastic bag, labeled, and cool-stored until being transferred to the laboratory. Under a dissection microscope, the emergence holes on leaf veins of *H. vitis* and *Z. rhamni* nymphs (hatched eggs) and *A. atomus* adults (parasitized eggs) were counted [60]. Specifically, the emergence hole of the parasitoid from the vein is circular and slightly browned, while the exit hole of the hatched nymph has an elongated shape. It was not possible to distinguish the exit holes of the two leafhopper species. The percentage of parasitized eggs was also calculated.

### 2.4. Data Analysis

To examine the data, general linear models with ANOVA were performed with RStudio software version 2023.06.1+524 (packages used for the analysis are ‘lme4’ and ‘lmerTest’) [61] considering separately the three years of study.

For the captures with yellow sticky traps and drop cloth, ‘lmer’ function was performed in which time was the within-subjects factor, while treatments (i.e., AM and PT) and clones (i.e., 297 and R3) were the between-subjects factors; interaction clone × treatment was also calculated. To satisfy ANOVA assumptions, data collected in each plot were transformed into the log (x + 1).

For leafhopper nymphs, a two-way ANOVA was performed with treatments (i.e., AM and PT) and clones (297 and R3) as factors, after transforming the nymphs recorded over time in each plot in the parameter “cumulative leafhopper-days” due to the presence of too many dates with zero individuals in most plots.

This parameter was calculated by following the equation from Ruppel [62]:

Arthropod-days = (X_i+1_ − X_i_) × [Y_i_ + Y_i+1_)/2] (1)

where, for each taxon, X_i_ and X_i + 1_ were two adjacent sampling dates, and Y_i_ and Y_i + 1_ were the number of individuals counted in these adjacent sampling dates. The cumulative arthropod-days were computed by sequentially summing the individual arthropod-days. This parameter represents well the cumulative physiological injury caused by the leafhoppers.

For leafhopper nymphs and adults, the data of each sampling date were analyzed with a two-way ANOVA considering treatments (i.e., AM and PT) or clones (i.e., 297 and R3) as factors. To satisfy ANOVA assumptions, the number of nymphs per plot and the captures per trap were transformed into square-root.

For leafhopper ovipositions (total, hatched, and parasitized eggs) and the percentage of parasitized eggs, a two-way ANOVA was performed with treatments (i.e., AM and PT) and clones (i.e., 297 and R3) as factors. To satisfy ANOVA assumptions, the number of total leafhopper eggs and the percentage of parasitized leafhopper eggs were transformed into the square root and arcsine, respectively.

Within spiders, the relative proportion of the two hunting-strategy groups, i.e., hunters and web-builders, was compared using a G-test of goodness of fit (also called the likelihood ratio test).

## 3. Results

### 3.1. Sampled Arthropods

All leafhopper nymphs (N. 1497) recorded on leaves and adults (N. 118,187) captured on yellow sticky traps belonged to *H. vitis* and *Z. rhamni*, while no specimen of *E. vulnerata* was recorded. *Hebata vitis* was more abundant than *Z. rhamni* with 82% of the nymphs and 91% of the adults belonging to the former species.

The three considered taxa of hymenopteran parasitoids captured on yellow sticky traps, i.e., *A. atomus*, Ichneumonoidea, and Chalcidoidea excl. Mymaridae, represent, respectively, 26%, 70%, and 4% of the total (N. 15,022). The total number of spiders captured with the drop cloth and yellow sticky traps was similar (N. 1768 and N. 1980, respectively). Spiders collected with the drop cloth belonged to 13 families and 31 different taxa, with hunters being more abundant than web-builders (84% and 16%, respectively) (Table S3 in the Supplementary Materials). At the family level, Oxyopidae was the dominant (59%), followed by Thomisidae (12%) and Araneidae (10%).

### 3.2. Populations of Grapevine Leafhoppers and Their Natural Enemies

#### 3.2.1. *Hebata vitis* Nymphs on Leaves

**Figure 2:**
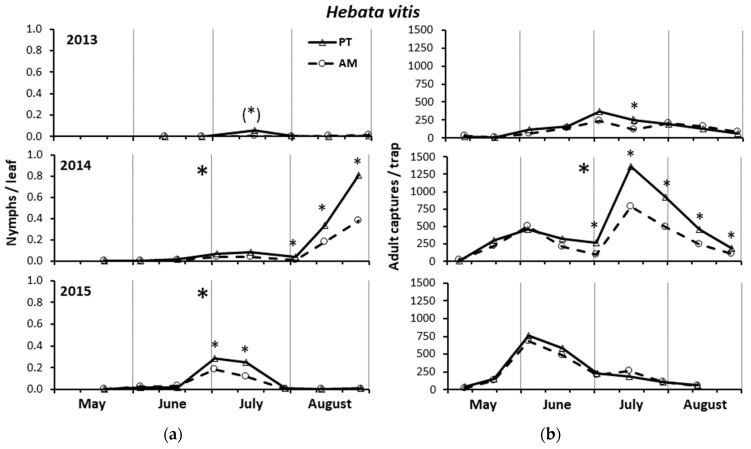
Densities of *Hebata vitis* nymphs (**a**) and adults (**b**) recorded from May to August in the three years of study in the alternate mowing and periodical tillage treatments (AM and PT, respectively). The asterisks in the top center of each graph indicate the years in which the total nymphs (**a**) and adults (**b**) significantly differed between the treatments (see ANOVA reported in Results). The asterisks in correspondence with each date indicate the significant differences between treatments in that specific sampling. * = significant differences at 0.05 using ANOVA; (*), i.e., asterisk in brackets = difference close to the significance level (*p* = 0.065).

In 2013, the *H. vitis* infestation level was negligible with a maximum of 0.055 nymphs per leaf in PT in mid-July in coincidence with the second generation (Figure 2a). The accumulated nymphs were higher in PT than AM (by 2.4×) and clone 297 than clone R3 (by 9.3×), but the differences did not reach the level of significance for either treatment (PT = 117.5 ± 65.1, AM = 36.0 ± 22.6; F = 2.75; d.f. = 1, 4 *p* = 0.173) or clone (clone 297 = 138.5 ± 57.1, clone R3 = 15.0 ± 7.6; F = 6.31; d.f. = 1, 4; *p* = 0.066). Nevertheless, referring to the single sampling dates, the nymph population was marginally significantly higher in PT than AM in mid-July (16 July, F = 6.35; d.f. =1, 4; *p* = 0.065) (Figure 2a).

In 2014, the *H. vitis* infestation level reached a maximum of approximately one nymph per leaf in PT in late August in coincidence with the third generation (Figure 2a). The accumulated *H. vitis* nymphs were significantly higher in PT than AM (PT = 1246.3 ± 132.0, AM = 595.3 ± 55.4; F = 36.80; d.f. = 1, 4; *p* = 0.004) (by 2.1×), whereas the influence of the clone was not significant (clone 297 = 785.5 ± 166.8, clone R3 = 1056.0 ± 226.1; F = 6.35; d.f. = 1, 4; *p* = 0.065). Specifically, the nymph population was significantly greater in PT than AM from late July to late August (31 July, F = 8.97. d.f. = 1, 4; *p* = 0.040; 12 August, F = 20.08; d.f. = 1, 4; *p* = 0.011; 25 August, F = 9.78; d.f. = 1, 4; *p* = 0.035) (Figure 2a). Referring to a single sampling date, the population density was significantly greater in clone R3 than in clone 297 in mid-August (12 August, F = 45.09; d.f. = 1, 4; *p* = 0.003).

In 2015, the *H. vitis* infestation level reached a maximum of approximately 0.3 nymphs per leaf in PT in July in coincidence with the second generation (Figure 2a). The accumulated nymphs were significantly higher in PT than AM (PT = 770.4 ± 49.7, AM = 473.4 ± 26.6; F = 23.60; d.f. = 1, 4; *p* = 0.008) (by 1.6×), whereas the influence of the clone was not significant (clone 297 = 596.4 ± 87.5, clone R3 = 647.4 ± 98.9; F = 0.70; d.f. = 1, 4; *p* = 0.45). Referring to the sampling dates, the nymph population was significantly greater in PT than AM in early and mid-July (1 July, F = 9.37. d.f. = 1, 4; *p* = 0.038; 13 July, F = 16.21; d.f. = 1, 4; *p* = 0.016) (Figure 2a) and in clone R3 than clone 297 in early June (3 June, F = 20.38. d.f. = 1, 4; *p* = 0.011).

#### 3.2.2. *Hebata vitis* Adult Captures by Yellow Sticky Traps

In 2013, the *H. vitis* captures were not significantly influenced by either treatment (F = 1.25; d.f. = 1, 60; *p* = 0.268) or clone (F = 0.01; d.f. = 1, 60; *p* = 0.914). However, in mid-July, significantly higher captures were recorded in PT than AM (16 July, F = 10.97; d.f. = 1, 4; *p* = 0.030) (Figure 2b).

In 2014, the captures were significantly higher in PT than AM (F = 18.43; d.f. = 1, 60; *p* < 0.0001) (by 1.6×), with significant differences from early July to late August (1 July, F = 52.05; d.f. = 1, 4; *p* = 0.002; 15 July, F = 18.26; d.f. = 1, 4; *p* = 0.013; 31 July, F = 44.53; d.f. = 1,4; *p* = 0.003; 12 August, F = 56.73; d.f. = 1, 4; *p* = 0.002; 25 August, F = 16.50; d.f. = 1,4; *p* = 0.015) (Figure 2b). No significant differences were recorded in the adult population between the two clones (F = 0.34; d.f. =1, 60; *p* = 0.562), although, in mid-August, significantly greater captures were recorded in clone R3 than clone 297 (12 August, F = 24.61; d.f. = 1, 4; *p* = 0.008).

In 2015, the captures were not significantly influenced by either treatment (F = 2.02; d.f. = 1, 53; *p* = 0.161) or clone (F = 0.85; d.f. = 1, 53; *p* = 0.362) (Figure 2b).

#### 3.2.3. *Zygina rhamni* Nymphs on Leaves

**Figure 3:**
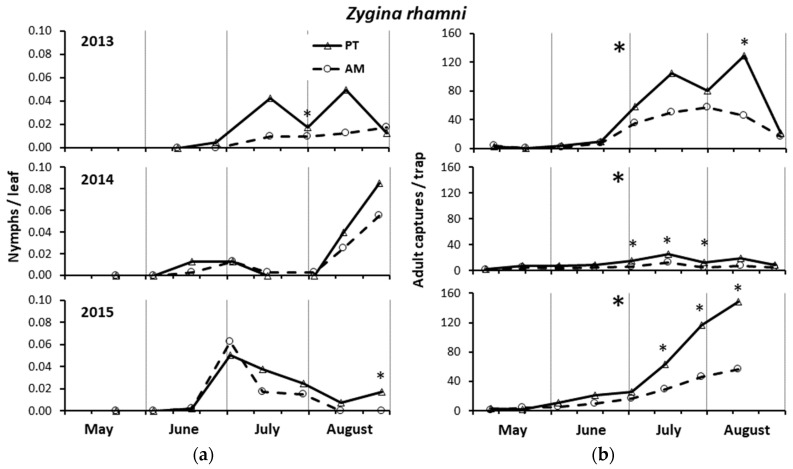
Densities of *Zygina rhamni* nymphs (**a**) and adults (**b**) recorded from May to August in the three years of study in the alternate mowing and periodical tillage treatments (AM and PT, respectively). The asterisks in the top center of each graph indicate the years in which the total nymphs (**a**) and adults (**b**) significantly differed between the treatments (see ANOVA reported in Results). The asterisks in correspondence with each date indicate the significant differences between treatments in that specific sampling. * = significant differences at 0.05 using ANOVA.

In 2013, the *Z. rhamni* infestation level was low with a maximum of 4.4 and 5 nymphs on 100 leaves in PT in mid-July and late August in coincidence with the second and third generations, respectively (Figure 3a). On average, the accumulated nymphs were higher in PT than AM (by 2.5×) but the differences did not reach the level of significance (PT = 184.3 ± 107.6, AM = 62.8 ± 27.8; F = 2.33; d.f. = 1, 4; *p* = 0.20). Referring to the single sampling date, the nymph infestation was significantly higher in PT than AM in late July (30 July, F = 9.15; d.f. = 1, 4; *p* = 0.039). In clone 297, the accumulated nymphs were six times higher than in clone R3 with marginally significant differences (clone 297 = 221.5 ± 91.2, clone R3 = 25.5 ± 9.7; F = 6.07; d.f. = 1, 4; *p* = 0.069), which, however, were significant on one sampling date (16 July, F = 34.79; d.f. = 1, 4: *p* = 0.004; 30 July, F = 23.62; d.f. = 1, 4; *p* = 0.008).

In 2014, the *Z. rhamni* infestation level was very low, with a maximum of one nymph on 100 leaves in PT in late August, coinciding with the third generation (Figure 3a). The accumulated nymphs were not significantly influenced by either treatment (PT = 148.6 ± 10.0, AM = 97.4 ± 21.3; F = 4.84; d.f. = 1, 4; *p* = 0.093) or clone (clone 297 = 132.8 ± 15.9, clone R3 = 113.2 ± 26.1; F = 0.70; d.f. = 1, 4; *p* = 0.450). However, the population density was significantly higher in clone 297 than clone R3 in early July (1 July, F = 22.48; d.f. = 1, 4; *p* = 0.009).

In 2015, the *Z. rhamni* infestation level was low, with a maximum of 6.3 nymphs on 100 leaves in AM in early July, coinciding with the second generation (Figure 3a). The accumulated nymphs were not significantly influenced by either treatment (PT = 179.4 ± 48.8, AM = 130.1 ± 20.9; F = 0.80; d.f. = 1, 4; *p* = 0.42) or clone (clone 297 = 174.6 ± 52.2, clone R3 = 134.9 ± 15.1; F = 0.52; d.f. = 1, 4; *p* = 0.51). However, in late August, the nymph populations were significantly higher in PT than AM (26 August, F = 262.16; d.f. = 1, 4; *p* < 0.0001) and clone 297 than clone R3 (26 August, F = 13.01; d.f. = 1, 4; *p* = 0.023).

#### 3.2.4. *Zygina rhamni* Adult Captures by Yellow Sticky Traps

In 2013, the *Z. rhamni* captures were significantly higher in PT than AM (F = 9.44; d.f. = 1, 60; *p* = 0.003) (by 1.3×) and significantly greater in clone 297 than clone R3 (F = 4.33; d.f. = 1, 60; *p* = 0.042) (by 1.5×). Referring to the single sampling date, the captures were significantly higher in PT than AM in mid-August (13 August, F = 10.25; d.f. = 1, 4; *p* = 0.033) (Figure 3b) and in clone 297 than clone R3 in early May and mid-August (9 May, F = 12.89; d.f. = 1, 4; *p* = 0.023; 13 August, F = 10.06; d.f. = 1, 4; *p* = 0.034).

In 2014, the captures were significantly higher in PT than AM (F = 30.63; d.f. = 1, 60; *p* < 0.0001) (by 2.5×), but no significant differences were recorded between clones (F = 0.69; d.f. = 1, 60; *p* = 0.410). Referring to the single sampling dates, the captures were significantly higher in PT than AM in July (1 July, F = 18.16; d.f. = 1, 4; *p* = 0.013; 15 July, F = 13.98; d.f. = 1, 4; *p* = 0.020; 31 July, F = 8.69; d.f. = 1, 4; *p* = 0.042) (Figure 3b).

In 2015, the captures were significantly higher in PT than AM (F = 31.35; d.f. = 1, 53; *p* < 0.0001) (by 2.3×), with significant differences from mid-July to mid-August (13 July, F = 39.07; d.f. = 1, 4; *p* = 0.003; 28 July, F = 23.89; d.f. = 1, 4; *p* = 0.008; 11 August, F = 21.09; d.f. 1, 4; *p* = 0.010) (Figure 3b). No significant differences were observed between clones (F = 0.17; d.f. = 1, 53; *p* = 0.675), although clone 297 was significantly more infested than clone R3 in early May and mid-July (8 May, F = 17.67; d.f. = 1, 4; *p* = 0.001; 14 July, F = 9.24; d.f. = 1, 4; *p* = 0.038).

### 3.3. Leafhopper Eggs and Anagrus atomus Activity

In 2013, there were less than 10 leafhopper eggs counted on leaves.

**Figure 4:**
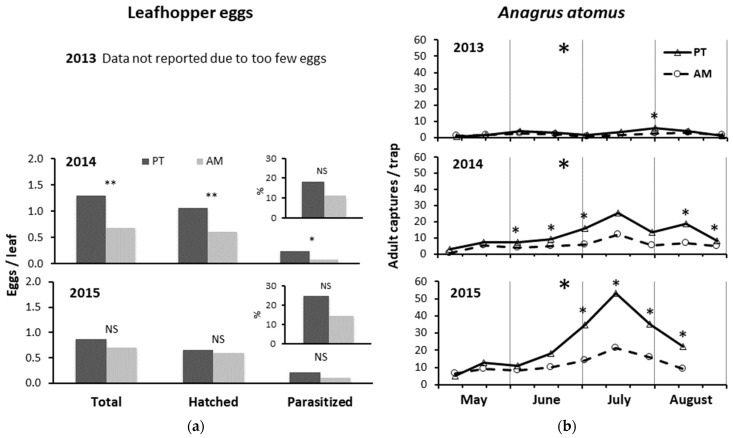
(**a**) Leafhopper eggs per leaf (total, hatched, and parasitized by *Anagrus atomus*) counted in the laboratory and percentage of those parasitized (%). (**b**) Densities of *Anagrus atomus* recorded on yellow sticky traps from May to August in the three years of study in the alternate mowing and periodical tillage treatments (AM and PT, respectively). In (**a**), * and ** = significant differences at 0.05 and 0.01, respectively, and NS = not significant, using ANOVA. In (**b**), the asterisks in the top center of each indicate the years in which the total captures significantly differed between the treatments (see ANOVA reported in Results). The asterisks in correspondence with each date indicate the significant differences between treatments in that specific sampling.

In 2014, the number of eggs, total, hatched, and parasitized, was significantly higher in PT than AM (total: F = 50.00; d.f. = 1, 4; *p* = 0.002; hatched: F = 44.16; d.f. = 1, 4; *p* = 0.003; parasitized: F = 8.89; d.f. = 1, 4; *p* = 0.041), but the percentage of parasitized eggs did not significantly differ between the two treatments (F = 4.42; d.f. = 1, 4, *p* = 0.103) (Figure 4a). The number of eggs, total and hatched, was significantly higher in clone R3 than clone 297 (total eggs: F = 8.00; d.f. = 1, 4; *p* = 0.047; hatched eggs: F = 44.16; d.f. = 1, 4; *p* = 0.003). However, there were no significant differences between the two clones, either for the number of parasitized eggs (F = 11.53; d.f. = 1, 4; *p* = 0.829), or their percentage (F = 0.05; d.f. = 1, 4; *p* = 0.841).

In 2015, either the number of eggs, as total, hatched, and parasitized, or the percentage of those parasitized were not significantly different between the two treatments (total: F = 1.20; d.f. = 1, 4; *p* = 0.340; hatched: F = 0.21; d.f. = 1, 4; *p* = 0.674; parasitized: F = 3.20; d.f. = 1, 4; *p* = 0.146; % of parasitized: F = 3.24; d.f. = 1, 4; *p* = 0.150) (Figure 4a). Moreover, there were no significant differences between the two clones (total eggs: F = 0.07; d.f. = 1, 4; *p* = 0.940; hatched eggs: F = 0.05; d.f. = 1, 4; *p* = 0.832; parasitized eggs: F = 0.04; d.f. = 1, 4; *p* = 0.851; % of parasitized: F = 0.11; d.f. = 1, 4; *p* = 0.755).

### 3.4. Hymenopteran Parasitoids Captures by Yellow Sticky Traps

In all three years, the captures of *A. atomus* adults were significantly higher in PT, where higher leafhopper infestations were observed, than in AM (2013: F = 4.88; d.f. = 1, 60; *p* = 0.031; 2014: F = 47.38; d.f. = 1, 60; *p* < 0.0001; 2015: F = 39.17; d.f. = 1, 53; *p* < 0.0001) (by 1.4×, 2.1×, and 2.0×, respectively) (Figure 4b). Referring to the sampling dates, the captures were significantly higher in PT than AM in one case in 2013 (30 July, F = 21.56; d.f. = 1, 4; *p* = 0.010), in five cases in 2014 (3 June, F = 9.23; d.f. = 1, 4; *p* = 0.039; 17 June, F = 11.02; d.f. = 1, 4; *p* = 0.029; 1 July, F = 60.03; d.f. = 1, 4; *p* = 0.001; 12 August, F = 26.82; d.f. = 1, 4; *p* = 0.007; 25 August, F = 9.47; d.f. = 1, 4; *p* = 0.037), and in four cases in 2015 (1 July, F = 104.83; d.f. = 1, 4; *p* = 0.001; 3 July, F = 45.51; d.f. = 1; 4; *p* = 0.003; 28 July, F = 25.38; d.f. = 1, 4; *p* = 0.007; 11 August, F = 12.35, d.f. = 1, 4; *p* = 0.025) (Figure 4b). The captures were not significantly different between the two clones (2013: F = 3.89; d.f. = 1, 60; *p* = 0.053; 2014: F = 0.115; d.f. = 1, 60; *p* = 0.736; 2015: F = 0.0002; d.f. = 1, 60; *p* = 0.988), although the captures were significantly higher on clone 297 than clone R3 in early June 2013 (4 June, F = 37.31; d.f. = 1, 4; *p* = 0.004).

**Figure 5:**
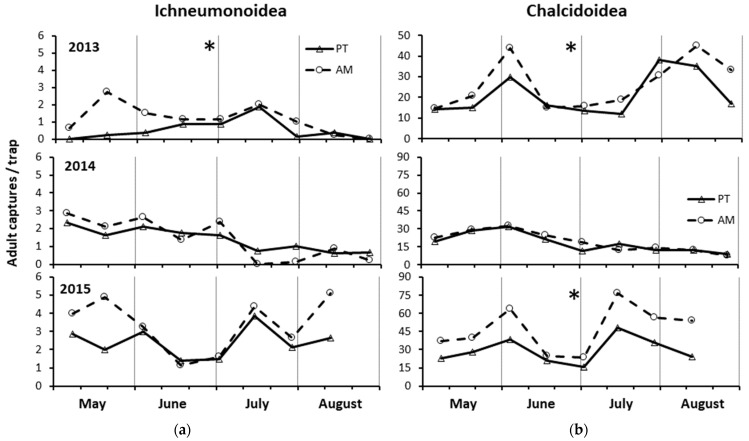
Densities of (**a**) Ichneumonoidea and (**b**) Chalcidoidea excl. Mymaridae recorded on yellow sticky traps from May to August in the three years of study in the alternate mowing and periodical tillage treatments (AM and PT, respectively). The asterisks in the top center of each graph indicate, for each taxon, the years in which the total captures significantly differed between the treatments (see ANOVA reported in Results).

In 2013, a significantly higher amount of Ichneumonoidea was captured in AM than PT (F = 13.29; d.f. = 1, 60; *p* = 0.0006) (by 2.2×) (Figure 5a). The captures were not significantly different between the two clones (F = 0.99; d.f. = 1, 60; *p* = 0.32). In 2014, the captures were similar either between the two treatments (F = 0.01; d.f. = 1, 60; *p* = 0.928) and clones (F = 3.73; d.f. = 60; *p* = 0.058). In 2015, the captures were not significantly different between treatments (F = 1.49; d.f. = 1, 53; *p* = 0.228), while they were between the clones, being greater in clone R3 compared to clone 297 (F = 6.88; d.f. = 1, 53; *p* = 0.011) (by 1.5×).

In 2013, a significantly higher amount of Chalcidoidea excl. Mymaridae was recorded in AM than PT (F = 11.17; d.f. = 1, 60; *p* = 0.0014) (by 1.3×) and in clone R3 than clone 297 (F = 10.86; d.f. = 1, 60; *p* = 0.0017) (by 1.3×) (Figure 5b). In 2014, the population level was similar between the two treatments (F = 0.01; d.f. = 1, 60; *p* = 0.934) and significantly higher in clone R3 than clone 297 (F = 0.06; d.f. 1. 60; *p* = 0.036) (by 1.1×). In 2015, the captures were significantly higher in AM than PT (F = 46.01; d.f. = 1, 53; *p* < 0.0001) (by 1.7×), while their amount did not differ between the clones (F = 0.38; d.f. = 1, 53; *p* = 0.539).

### 3.5. Spiders by Drop Cloth

**Figure 6:**
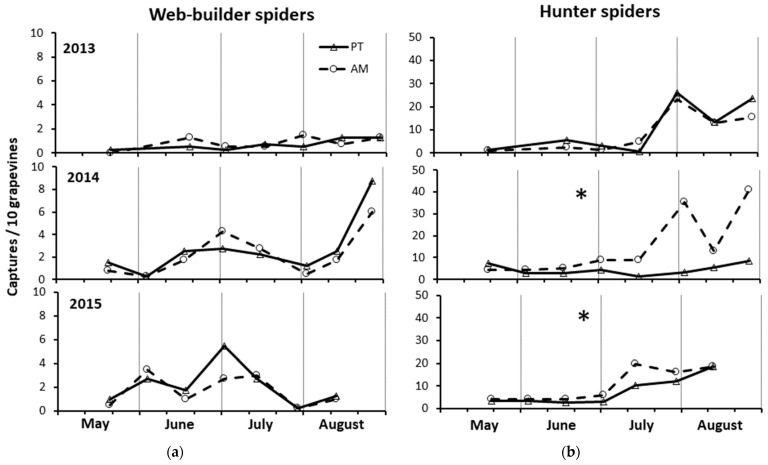
Densities of spiders, web-builders (**a**) and hunters (**b**), recorded by drop cloth method from May to August in the three years of study in the alternate mowing and periodical tillage treatments (AM and PT, respectively). The asterisks in the top center of each graph indicate, for each hunting group, the years in which the total captures significantly differed between the treatments (see ANOVA reported in Results).

In none of the three years, the captures of web-builder differed between treatments (2013: F = 0.48; d.f. = 1, 46; *p* = 0.491; 2014: F = 1.31; d.f. = 1, 53; *p* = 0.258; 2015: F = 0.58; d.f. = 1, 46; *p* = 0.449) and clones (2013: F = 0.15; d.f. = 1, 46; *p* = 0.699; 2014: F = 0.35; d.f. = 1, 53; *p* = 0.559; 2015: F = 3.33; d.f. = 1, 46; *p* = 0.075) (Figure 6a).

In 2013, the population density of hunters did not differ either between treatments (F = 0.99; d.f. = 1, 46; *p* = 0.323) or between clones (F = 2.12; d.f. = 1, 46; *p* = 0.152) (Figure 6b). In the following two years of study, a significantly greater number of hunters was captured in AM than PT (2014: F = 29.25; d.f. = 1, 53; *p* < 0.0001; 2015: F = 4.38; d.f. = 1, 46; *p* = 0.042) (by 3.4× and 1.4×, respectively). This occurred throughout the two sampling seasons, except in the mid-May sampling date of 2014 (Figure 6b). The clone did not influence hunter captures even in the two years of 2014 and 2015 (2014: F = 1.12; d.f. = 1, 53; *p* = 0.732; 2015: F = 0.98; d.f. = 1, 46; *p* = 0.327).

The total number of hunters was more than 5.4× higher compared to that of web-builders in all years (93% in 2013, 80% in 2014, and 82% in 2015), with the percentage of hunters vs. that of web-builders being significantly higher in 2013 than in 2014 and 2015 (G-test, *p* < 0.0001). Furthermore, the total number of hunters, captured in the three years of 2013–2015, was significantly higher than that of web builders in both AM and PT (by 7.1× and 3.9×, respectively) (G-test, hunters in AM and PT, 87.6% and 79.6%, respectively, *p* < 0.0001). However, the percentages of hunters on the total number of spiders significantly differed between the two treatments only in 2014 and 2015 (G-test, 2013: AM = 91.4% and PT = 94.0%, *p* = 0.16; 2014: AM = 87.0% and PT = 62.0%, *p* < 0.0001; 2015: AM = 85.9% and PT = 77.8%, *p* = 0.005).

### 3.6. Spiders by Yellow Sticky Traps

**Figure 7:**
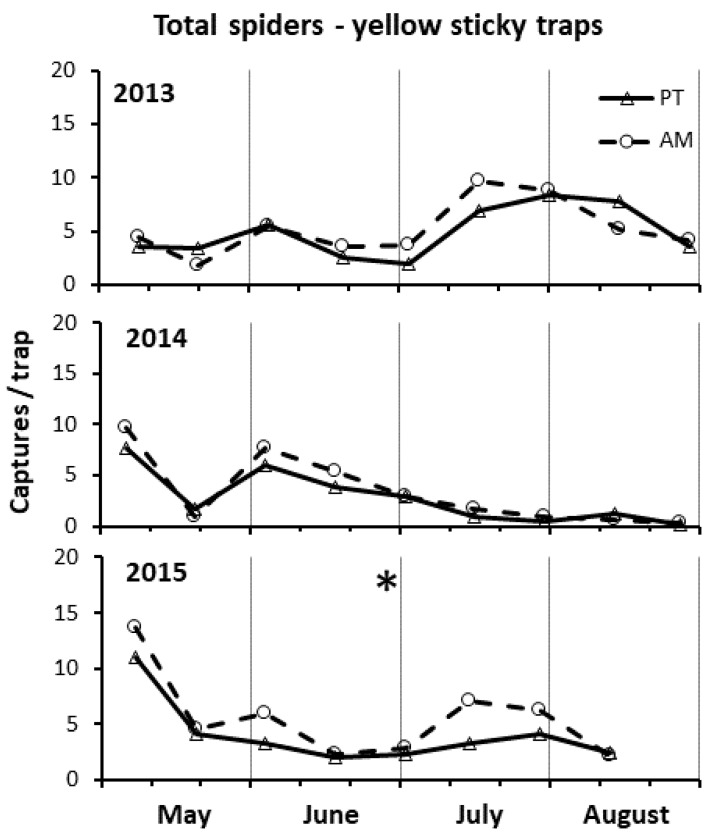
Densities of total spiders on yellow sticky traps from May to August in the three years of study in the alternate mowing and periodical tillage treatments (AM and PT, respectively). The asterisk in the top center of the graph indicates that, in 2015, the total captures significantly differed between the treatments (see ANOVA reported in Results).

In 2013, spider captures were not significantly different between the two treatments (F = 0.33; d.f. = 1, 60; *p* = 0.568), although they were higher in AM than PT from mid-June to late July (Figure 7). Spider captures were significantly higher in clone R3 than clone 297 (F = 5.32; d.f. = 60; *p* = 0.025) (by 1.3×). In 2014, spider captures were not significantly different between the two treatments (F = 1.03; d.f. = 1, 60; *p* = 0.315), although they were higher in AM than PT in early May and the June–July period (Figure 7). In 2015, spider captures were significantly higher in AM than PT (F = 7.68; d.f. = 1, 53; *p* = 0.008) (by 1.4×). Both in 2014 and 2105, the spider captures did not differ between clones (2014: F = 0.24; d.f. = 1, 60; *p* = 0.623; 2015: F = 0.83; d.f. = 1, 53; *p* = 0.37).

### 3.7. Interaction between Treatments and Cultivar Clone

No significant interactions between the treatment and clone were found for either leafhoppers or their natural enemies.

## 4. Discussion

### 4.1. Influence of Year on Leafhopper Abundance

The fact that, in 2013, *H. vitis* nymph populations were much lower than in the two following years (approximately 12× and 8× lower than in 2014 and 2015, respectively) may be associated with the scarcity of rainfall that occurred in that year (see Table S2 in the Supplementary Materials). It is known that moderate water stress can cause a reduction in *H. vitis* infestation [46] due to egg mortality [47]. The effect was more evident in the alternate mowing plots (lower in 2013 than in 2014 and 2015, approximately by 17× and 13×, respectively) than in the periodical tillage ones (lower in 2013 than in 2014 and 2015, approximately by 11× and 7×, respectively). This suggests that the scarcity of rainfall could have caused the higher mortality of eggs in the alternate mowing plots where the competition for water between the grapevine and the herbaceous vegetation could have accentuated the state of water stress. Moreover, for *Arboridia kermanshah* Dlabola (Hemiptera: Cicadellidae), it was shown that the leafhopper population density, pest damage percentage, and egg parasitism were higher in the normal irrigation plots than in plots with minimal irrigation [63]. The increased density of leafhoppers [*Erythroneura elegantula* Osborn and *Erasmoneura variabilis* (Beamer); Hemiptera: Cicadellidae] was observed in well-irrigated grapevines [64].

In a Sardinian (Italy) vineyard, the population level of leafhoppers *Jacobiasca lybica* (Berg. & Zan) (Hemiptera: Cicadellidae) and *Z. rhamni* were not significantly different between tilled soil and natural ground cover [65], although this result could be associated with the fact that, during summer, the resident vegetation dried up and did not compete with the grapevine for water and nutrients.

### 4.2. Influence of Inter-Row Management on Leafhopper Abundance and Anagrus atomus Activity

This study showed that the densities of *H. vitis* and Z. *rhamni* nymphs on the grapevine canopy were lower in the alternate mowing plots than in those periodically tilled. The differences reached statistically significant levels only in the first year of study (i.e., 2013) for *Z. rhamni*, and in the second and third years of study for *H. vitis* (i.e., 2014 and 2015). In agreement with these data, significantly lower *Erythroneura* spp. leafhopper densities were recorded in Californian vineyards with native grasses versus those with bare soil [45,66].

Moreover, the number of leafhopper eggs, recorded on the grapevine leaves in late summer in 2014 and 2015, was lower in the alternate mowing than in the periodically tilled treatment, however, reaching a significant level only in 2014. For each treatment, the total amount of leafhopper eggs can be prevalently assigned to *H. vitis* (ratio *H. vitis*/*Z. rhamni* nymphs for 2014: 6.1 in AM and 8.3 in PT; and, for 2015: 3.6 in AM and 4.3 in PT). However, to explain the difference between treatments on leafhopper densities, it is necessary to consider at least two factors, namely, the preferred site for laying eggs by leafhopper females and the activity of natural enemies, in particular the leafhopper egg parasitoid *A. atomus* and the generalist predators (e.g., spiders) of leafhoppers nymphs and adults. The two aspects are discussed below.

#### 4.2.1. Egg-Laying Preference

For *H. vitis*, it is already known that a greater grapevine vigor is associated with a higher nymph population level [43,44] because egg laying is favored [31,33]. Similarly, Wilson et al. [67] found that the reduced egg laying and nymph abundance for *E. elegantula* at the vineyard edge were primarily due to a reduced vigor of these grapevines compared with the interior part of the vineyard. For this purpose, in the vineyard under study, in the period 2013–2015, a reduction in grapevine vigor was detected in the alternate mowing plots versus periodically tilled ones [20]. Consequently, the differences in oviposition between the treatments may have been favored because that leafhopper adults, due to the proximity of the plots, could have been attracted by the more vigorous grapevine in tilled plots. However, in both 2014 and 2015, the differences between the two treatments were greater for ovipositions than for female captures (in 2014, the ratio PT/AM for ovipositions was 1.92 and for female captures 1.67; and, in 2015, the ratio PT/AM for ovipositions was 1.23 and for female captures 0.97), suggesting that the number of eggs per female was lower in the alternate mowing plots than in the periodical tillage plots. It can be assumed that, given the same density of females, their fecundity increases with the increase in vigor of the grapevines. Considering nymph infestation, the differences in population levels between periodical tillage and alternate mowing increased by 2.02× in 2013, 1.30× in 2014, and 1.77× in 2015 compared to the differences in adult captures, further demonstrating that infestation levels were not proportional to the number of females that oviposited. Therefore, the higher nymph population levels in periodical tillage compared to alternate mowing cannot be explained only by the greater attraction of adults to the periodic tillage plots, but effects on female fecundity and nymph mortality must also be considered.

#### 4.2.2. Activity of Natural Enemies

The differences in the leafhopper populations between the two inter-row management strategies could also be due to a different level of biological control by the egg parasitoid *A. atomus* and nymph predators. The data collected in the present study not only excluded a higher level of leafhopper egg parasitism in the alternate mowing plots but, on the contrary, highlighted a higher level of parasitism in the periodical tillage plots. Similarly, other studies reported that the parasitism rate of *Erythroneura* spp. was significantly higher in tilled than green-covered inter-rows [45,66]. Thus, the *A. atomus* activity was not directly influenced by the different inter-row management but appeared to be the density-dependent response to a different leafhopper egg amount between the two treatments. A close relationship between the *Erythroneura* leafhopper and *Anagrus* spp. density was also demonstrated by Costello et al. [68]. Furthermore, the nectar availability in alternate mowing is not essential for egg maturation in *A. atomus* [69].

The data collected in this study, while showing that alternate mowing did not favor the activity of *A. atomus*, suggest that this inter-row management is associated with the greater mortality of the leafhopper nymphs. In fact, the differences between the two treatments are lower for hatched eggs than for nymphs (in 2014, the ratio PT/AM for eggs was 1.77 and for nymphs 2.09; and, in 2015, the ratio PT/AM for eggs was 1.08 and for nymphs 1.63), suggesting a higher nymph mortality in alternate mowing plots. This increase in mortality is likely associated with nymph predation, although other mortality factors (e.g., increased exposure to sunlight or rain) cannot be excluded. In the present study, among potential predators of leafhopper nymphs, *Chrysoperla carnea* s.l. (Stephens) (Neuroptera: Chrysopidae) and spiders [39,70] were recorded. However, *C*. *carnea* captures on yellow sticky traps were negligible and did not differ between treatments (2013: PT = 11 and AM = 13; in 2014: PT = 4 and AM = 4; 2015: PT = 8 and AM = 12). In contrast, the data on spiders may be consistent with a different predation rate between the two treatments (see the next subsection ‘Effect of inter-row management on spider abundance’).

### 4.3. Effect of Inter-Row Management on Spider Abundance

The abundance of spiders, specifically the hunters sampled with the drop cloth and the total spiders captured on yellow sticky traps, were higher in the alternate mowing plots than in the periodical tillage plots. The beneficial effects on hunters were absent in the first year of study and became significant from the second year. Moreover, for the total spiders captured on yellow sticky traps, the differences in favor of alternate mowing appeared from the second year, becoming significant in the third year. On the contrary, in a study carried out in Californian vineyards, green-covered inter-rows did not increase the spider population compared to tilled inter-rows [66]. However, by comparing the spider abundance at the ground level in tilled and green-covered inter-rows, herbaceous vegetation enhances spider communities in vineyards [71,72,73].

In this study, the greater number of hunters on grapevines in the alternate mowing plots occurred despite the lower plant vigor. This result indirectly agrees with Pennington et al. [74] and Cargnus et al. [75], who showed that a reduction in leaf density due to pruning did not affect the abundance of spiders.

The greater leafhopper nymph mortality in alternate mowing compared to periodical tillage may be explained by the higher presence of hunting spiders. Indeed, the jumping spiders (Salticidae) and lynx spiders (*Oxyopes* spp.), recorded in high numbers in the present study, are reported as predators of *H. vitis* [39,75] and other Cicadellidae [76]. An association between the increase in spiders, as a consequence of cover crops in vineyard inter-rows, and leafhopper predation was reported for *E. elegantula* [77]. Under laboratory conditions, a high consumption of leafhoppers by lynx spider species (*Oxyopes javanus* Thorell) and *Salticus* sp. was recorded [78,79].

Spiders are known as predators of the European grapevine moth *Lobesia botrana* (Den. & Schiff.) (Lepidoptera Tortricidae) [48,80,81]. Although more hunters were present in the alternate mowing treatment than in the periodical tillage one, no significant influence on *L. botrana* infestation was observed (data reported in Bigot et al. [20]).

### 4.4. Effect of Inter-Row Management on Hymenopteran Parasitoids

The abundance of Ichneumonoidea and Chalcidoidea excl. Mymaridae on grapevines was higher in the alternate mowing treatment compared with the periodical tillage treatment with significant differences in some years. It suggests that the availability of pollen and nectar in inter-rows contributes to a greater occurrence of these parasitic wasps in the grapevine canopy. Our results are in agreement with other studies [40,82,83]. However, the higher presence of wasp parasitoids in alternate mowing, reported in the present study, did not significantly influence the *L*. *botrana* population (data reported in Bigot et al. [20]) in agreement with the study of Hoffmann et al. [84].

### 4.5. Effect of Clones on Leafhoppers and Natural Enemies

Although significant differences in leafhopper infestations between clones were observed in some years, the most infested clone was not always the same. For this reason, it is difficult to comment on this result. Moreover, the interactions between the treatment and clone were never statistically significant, showing that the clone did not interfere with the effect of inter-row management.

For the natural enemies, in some years, more abundant Ichneumonoidea (1 case) or Chalcidoidea excl. Mymaridae (2 cases) in clone R3 than clone 297 were observed. This phenomenon may be associated with the proximity of a meadow with flowering plants.

## 5. Conclusions

Although the influence of ground vegetation management on arthropods in vineyards has been studied in the past, the effect on grapevine leafhoppers, *H. vitis* and *Z. rhamni*, and their enemies, *A. atomus* and spiders, was first highlighted in the present study. Moreover, the findings emphasized the interaction between the effects on natural enemies and those on grapevine vigor recorded in the same vineyard and years. The population levels of *H. vitis* in vineyards are the results of several factors acting in succession, i.e., (i) the higher grapevine vigor, that favors greater leafhopper oviposition, (ii) the *A. atomus* egg parasitoid, whose activity is higher where there are more leafhopper eggs and, therefore, mitigates the effect of plant vigor, and (iii) the predators of the leafhopper nymphs such as hunting spiders.

By favoring the abundance of spiders on the grapevine canopy, the alternate mowing of the inter-rows in the vineyard represents a tool for increasing the predation of the grapevine leafhoppers. Although spiders, being generalist predators, may not be a key factor in the density-dependent regulation, their greater abundance may decrease the probability that leafhopper populations exceed the economic threshold.

The positive effect on leafhopper control of alternate mowing compared to periodical tillage adds to those on qualitative parameters and the sanitary status of bunches [20]. However, this inter-row management is suitable for quality viticulture as some of its benefits come at the expense of yield.

This strategy, particularly suitable for organic vineyards, should also be adopted in conventional vineyards due to both the benefits on production quality and the growing need for an alternative to pest chemical control.

## Data Availability

The data are available upon request.

## Supplementary Materials

The following supporting information can be downloaded at: https://www.mdpi.com/article/10.3390/insects15050355/s1, Table S1: Herbaceous plants recorded from May to August (2013–2015) in the study-vineyard plots belonging to alternate mowing treatment (AM). Classes of the inter-row coverage for each species: r = rare, less than 1.0%; 1 = 1.1–10.0%; 2 = 10.1–20.0%; and 3 = 20.1–40.0%. Labels in bold indicate plants in the flowering stage at the sampling moment; Table S2: Average monthly temperatures and monthly precipitation from May to September (2013–2015) obtained from a weather station (Gradisca d’Isonzo, Gorizia, ARPA FVG–OSMER, http://www.meteo.fvg.it/ (accessed on 15 February 2023)) located 6 km from the study vineyard; Table S3: Spiders collected with the drop-cloth method on grapevine canopy during three years in the study vineyard. NI = not identified to genus and species level.
