# Supplementary material for: Influence of Vineyard Inter-Row Management on Grapevine Leafhoppers and Their Natural Enemies
Source: Insects. 2024 May 14;15(5):355. doi: 10.3390/insects15050355 (PMC11122207; doi:10.3390/insects15050355)
Supplement: Supplementary file 1 [file insects-15-00355-s001.zip › insects-2957519-supplementary.pdf]

insects MDPI

Supplementary Materials

Table S1. Herbaceous plants recorded from May to August (2013–2015) in the study-vineyard plots belonging to alternate mowing treatment (AM). Classes of the inter-row coverage for each species: r = rare, less than 1.0%; 1 = 1.1–10.0%; 2 = 10.1–20.0%; and 3 = 20.1–40.0%. Labels in bold indicate plants in the flowering stage at the sampling moment.

Family	Species	May	June	Early July	Late July	August
Amaranthaceae	*Amaranthus deflexus* L.				r	
Amaranthaceae	*Amaranthus retroflexus* L.					**1**
Amaranthaceae	*Chenopodium album* L.				r	
Apiaceae	*Daucus carota* L.	2	1	**2**		
Apiaceae	*Pastinaca sativa* L.				**2**	
Asteraceae	*Ambrosia artemisiifolia* L.					1
Asteraceae	*Artemisia vulgaris* L.		2	1	2	1
Asteraceae	*Cirsium arvense* (L.) Scop.		1	r		
Asteraceae	*Crepis biennis* L.	**r**	**1**	**1**	1	**r**
Asteraceae	*Erigeron annuus* (L.)		2	**1**	**1**	**1**
Asteraceae	*Erigeron canadensis* L.	**2**	1	r	1	1
Asteraceae	*Helminthotheca echioides* (L.) Holub				1	**r**
Asteraceae	*Lactuca sativa* L.			r		
Asteraceae	*Matricaria chamomilla* L.	**r**				
Asteraceae	*Taraxacum* sect. *Taraxacum*	**r**		r		**r**
Brassicaceae	*Descurainia sophia* (L.) Webb ex Prantl				**r**	
Caprifoliaceae	*Valerianella locusta* L. (Laterr.)	**r**	**r**			
Caryophyllaceae	*Silene vulgaris* (Moench) Garcke			r		1
Caryophyllaceae	*Stellaria media* (L.) Vill.	**1**				
Convolvulaceae	*Convolvulus arvensis* L.	**r**	r	**1**		**1**
Equisetaceae	*Equisetum* sp.		r			
Euphorbiaceae	*Euphorbia verrucosa* L.	**r**	r			
Fabaceae	*Trifolium campestre* Cchreb.		**1**	r		**r**
Fabaceae	*Trifolium pratense* L.	**r**	r	**r**		**r**
Fabaceae	*Trifolium repens* L.	**1**	**1**	2		**1**
Fabaceae	*Vicia cracca* L.	**r**	r			
Geraniaceae	*Erodium cicutarium* (L.) L'Hér.	**1**	r			
Geraniaceae	*Geranium molle* L.	**r**	**r**	**r**		
Hypericaceae	*Hypericum perforatum* L.			**r**		r
Lamiaceae	*Lamium purpureum* L.	**r**				

Lamiaceae	*Mentha longifolia* (L.) L.	1		1		1
Papaveraceae	*Papaver rhoeas* L.	**1**				
Plantaginaceae	*Plantago lanceolata* L.			**1**		**r**
Plantaginaceae	*Plantago major* L.	**r**	r		1	r
Plantaginaceae	*Plantago media* L.				1	**1**
Plantaginaceae	*Veronica persica* Poir.	**1**	**r**			
Poaceae	*Cynodon dactylon* (L.) Pers.			**2**	1	**2**
Poaceae	*Dactylis glomerata* L.	**r**	1	**1**		
Poaceae	*Digitaria sanguinalis* (L.) Scop.		1			
Poaceae	*Hordeum murinum* L.	**3**	1		**r**	
Poaceae	*Lolium multiflorum* Lam.				**1**	
Poaceae	*Lolium pratense* (Huds.) Darbysh.			**1**		
Poaceae	*Poa annua* L.				r	**2**
Poaceae	*Poa pratensis* L.					**1**
Poaceae	*Setaria italica* (L.) P.Beauv.		1	r	3	**3**
Polygonaceae	*Polygonum aviculare* L.					**1**
Portulacaceae	*Portulaca oleracea* L.				1	1
Ranunculaceae	*Delphinium consolida* L.	**r**				
Ranunculaceae	*Fumaria officinalis* L.		3			
Solanaceae	*Solanum nigrum* L.					**1**
Urticaceae	*Urtica dioica* L.	**r**				
Violaceae	*Viola arvensis* Murray	**r**				

Table S2. Average monthly temperatures and monthly precipitation from May to September (2013–2015) obtained from a weather station (Gradisca d'Isonzo, Gorizia, ARPA FVG–OSMER, http://www.meteo.fvg.it/) located at 6 km from the study vineyard.

**Month**	**Average temperature (°C)**				**Rainfall (mm)**			
	**2013**	**2014**	**2015**	*****	**2013**	**2014**	**2015**	*****
May	16.3	17.2	19.0	17.7	343	93	80	112
June	21.5	21.7	22.8	21.6	76	122	110	105
July	25.9	22.2	26.5	23.7	28	182	88	94
August	24.3	22.0	24.9	23.6	73	124	182	102
September	19.3	18.5	19.7	18.9	204	112	162	172

* Mean value over a period (1990–2020)

Table S3. Spiders collected with the drop-cloth method on grapevine canopy during three years in the study vineyard. NI = not identified to genus and species level.

Family	Taxon	Hunting strategy	Total spiders
Araneidae	*Araneus* sp.	web-builder	48
Araneidae	Araneidae NI	web-builder	3
Araneidae	*Mangora acalypha* (Walckenaer)	web-builder	138
Araneidae	*Nuctenea umbricata* (Clerck)	web-builder	18
Clubionidae	*Clubiona* sp.	hunter	1
Gnaphosidae	*Drassodes* sp.	hunter	76
Gnaphosidae	*Micaria* sp.	hunter	6
Linyphiidae	*Lyniphia triangularis* (Clerck)	web-builder	7
Linyphiidae	Erigoninae NI	web-builder	61
Lycosidae	*Arctosa* sp.	hunter	6
Oxyopidae	*Oxyopes lineatus* Latreille	hunter	1112
Oxyopidae	*Oxyopes ramosum* Martini & Goeze	hunter	48
Philodromidae	*Philodromus aureoles* (Clerck)	hunter	14
Philodromidae	*Tibellus oblungus* (Walckenaer)	hunter	3
Pisauridae	*Pisaura* sp.	hunter	22
Salticidae	*Heliophanus* sp.	hunter	35
Salticidae	*Leptorchester berolinensis* C.L. Koch	hunter	8
Salticidae	*Philaeus chrysops* (Poda)	hunter	4
Salticidae	*Pseudicius encarpatus* (Walckenaer)	hunter	13
Salticidae	*Salticus* sp.	hunter	8
Salticidae	Salticidae NI	hunter	8
Sparassidae	*Micrommata virescens* (Clerck)	hunter	65
Theridiidae	*Episinus truncatus* Latreille	web-builder	1
Theridiidae	*Theridion* sp.	web-builder	20
Theridiidae	*Theridula gonyogaster* (Simon)	web-builder	2
Theridiidae	Theridiidae NI	web-builder	6
Thomisidae	*Runcinia grammica* (C.L. Koch)	hunter	39
Thomisidae	*Synema globosum* (Fabricius)	hunter	18
Thomisidae	*Thomisus onustus* Walckenaer	hunter	3
Thomisidae	*Xysticus* sp.	hunter	177
Uloboridae	*Uloborus walckenaerius* L.	web-builder	10
**Total spiders**			**1980**
